# Prevalence of Frailty and Associated Sociodemographic, Biomedical, and Biochemical Factors Amongst Participants Residing in Limpopo Province, South Africa

**DOI:** 10.3390/geriatrics10050134

**Published:** 2025-10-21

**Authors:** Reneilwe Given Mashaba, Kagiso P. Seakamela, Solomon S. R. Choma, Eric Maimela, Joseph Tlouyamma, Cairo Bruce Ntimana

**Affiliations:** 1 DIMAMO Population Health Research Centre, University of Limpopo, Sovenga St, Polokwane 0727, South Africapeacekagiso4@gmail.com (K.P.S.); joseph.tlouyamma@ul.ac.za (J.T.); 2 Department of Pathology, University of Limpopo, Sovenga St, Polokwane 0727, South Africa; solomon.choma@ul.ac.za; 3 Department of Public Health, Walter Sisulu University, Eastern Cape 5117, South Africa

**Keywords:** frailty, adults, Type 2 diabetes mellitus, dyslipidemia, obesity

## Abstract

**Background:** Frailty is a common syndrome amongst older individuals characterized by a progressive long-term loss of physical and or cognitive resilience. Given the high prevalence and chronic conditions and the lack of literature on frailty among rural older individuals in South Africa, the present study aimed to investigate the prevalence of frailty and its associated factors in older individuals residing in Limpopo province. **Methods:** This was a cross-sectional study, comprising 546 participants (48.4% males and 51.6% females) using Africa Wits-INDEPTH Partnership for Genomic Research (AWI-Gen) phase 2 data. Convenient sampling was used to select the participants. Frailty was measured using the five criteria proposed by Fried. Data was analyzed using Statistical Package for the Social Sciences (SPSS) 27. **Results:** The mean age (SD) of the participants was 66.78 ± 5.72. The proportion of individuals living with frailty was 26.4%. Individuals living with frailty were significantly older than both pre-frail and non-frail individuals. Current smokers significantly had higher proportion of frailty compared to both pre-frail and non-frail. The proportion of frailty reduced as the level of education increased. The present study found no association between biological sex and frailty. The likelihood of having frailty increased with age. On the unadjusted model, there was a significant association between frailty and 66 and above age group (OR: 1.61; 95% CI: 1.00–2.60). On the fully adjusted model the same age group was 1.75 more likely to be frail with a *p* value of 0.001. The present study found no significant association between marital status, smoking, alcohol status, current smoker, hypertension, diabetes, and obesity with frailty. Centrally obese participants were 0.48 and 0.37 times less likely to have frailty on unadjusted and adjusted models, respectively. Participants with dyslipidemia indicated by high total cholesterol (TC) were 2.25 times more likely to be associated with frailty. **Conclusions:** The prevalence of frailty was 26.4% and it was associated with age, educational status and dyslipidemia. Based on the findings of the present study, the authors recommend implementation of screening programs, for frailty in healthcare settings, especially targeting older adults with comorbidities.

## 1. Introduction

Frailty is a common syndrome amongst older individuals, characterized by a progressive long-term loss of physical and or cognitive resilience [1]. The British Geriatric Society defined frailty as a distinctive health state related to the aging process in which multiple body systems gradually lose their built-in reserves [2]. These definitions show that frailty is complicated and involves various systems, making older adults gradually lose their physical strength and resilience, making them more prone to health problems; thus motivating research on frailty among the elderly to evaluate frailty prevalence and risk factors to promote early detection and prevention, especially in under-researched African populations. The average life expectancy in South Africa is 66.87 years [63.50 years for males and 69.90 years for females] [3]. This is an increment from an average life expectancy of 62.88 years reported for the year 2023 [3]. The increment in life expectancy is attributed to improvements in life choices, access to healthcare, improved socio-economic status, and medical advancements [4,5]. Although an increment in life expectancy is generally considered a good thing, aging is a multifaceted process involving the simultaneous development of disease, functional limitations, and social vulnerability, factors associated with the possibility of an increased risk of developing physical frailty [6].

The prevalence of individuals living with frailty in South Africa was reported to be between 4 and 59.1%, depending on the population sampled and tools used [7,8]. The prevalence of frailty tends to be higher in individuals with chronic diseases and lifestyle conditions [9,10]. Previous studies conducted in the Ga-Dikgale study area reported a high prevalence of chronic diseases and lifestyle conditions such as diabetes (11%), hypertension (33%), obesity (35.0%), central obesity (59.9%), and HIV (17%) [11,12,13,14,15,16]. This may suggest that this population has a higher risk of developing physical frailty. The present study aimed to investigate the prevalence of frailty and its associated factors in older individuals residing in Limpopo province.

## 2. Materials and Methods

### 2.1. Design, Setting, Sampling, and Participants

This cross-sectional study included participants who participated in the phase 2 cohort of the African Wits INDEPTH Partnership for Genomic Research (AWI-Gen). The participants were selected using convenient sampling; participants with complete records to answer the aims, objectives of the present study were included, and those with incomplete records were excluded. The total number of participants was 1239. About 57 participants were excluded due to incomplete records, and 636 participants were excluded because they were aged less than 60 years, since they cannot be analyzed as a geriatric population. As a result, the study was conducted on 546 participants. Participants were recruited from villages that fall within the Ga-Dikgale tribal authority situated in the Capricorn district of Limpopo province, South Africa. This area is of low socio-economic status and its inhabitants mostly originate from the Ba-Pedi tribe. The Turfloop Research Ethics Committee (TREC) (REC-0310111-031) approved the study protocol. All the participants who took part in the study signed an informed consent form. Permission to conduct the study in Dikgale villages was sought from the Dikgale Tribal Authority.

### 2.2. Data Collection

On the day of participation, participants were brought to the Dikgale Mamabolo Mothiba (DIMAMO) Population Health Research Centre (PHRC), situated in the University of Limpopo. Written consent was obtained from the participants prior to enrollment. The research team used the local language (Sepedi) during the data collection process, and all questionnaires and consent forms were translated into the local language to ensure that participants who may not be proficient in English can fully comprehend the information presented to them. This ensured that the consent they gave was informed, and the answers they gave to the questionnaire were accurate.

All tests were performed following the AWI-Gen standard operation procedures (SOPs). Data was recorded into the Red Cap data collection tool. Blood samples were collected by a qualified research nurse and further processed and analyzed by certified laboratory technicians. Trained research assistants, following the AWI-Gen standard operation procedures, collected anthropometric measurements, frailty components, body composition, subcutaneous adipose tissue, and visceral adipose tissue. The questionnaire was used to solicit information relating to socioeconomic status (educational level, marital status, etc.), lifestyle factors (smoking and alcohol status). Biochemical parameters, including total cholesterol (TC), triglycerides (TG), high-density lipoprotein cholesterol (HDL-C), glucose, insulin, albumin-to-creatinine ratio (ACR), and estimated glomerular filtration rate (eGFR), were analyzed using a Randox Plus clinical chemistry analyzer (UK). The levels of TC, HDL-C, and TG were used to calculate low-density lipoprotein cholesterol (LDL-C) using the Friedewald formula, expressed in mmol/L: LDL-C = (TC) − (HDL-C) − (TG/5). However, this formula was not applied for TG concentrations exceeding 4.5 mmol/L More details included in the questionnaire were reported elsewhere [16,17,18].

### 2.3. Frailty Measurement

Frailty was evaluated according to the five criteria proposed by Fried [19], which included following the original cut-off points, with some adaptation. (1) Unintentional weight loss, (2) Self-reported exhaustion: Based on the question, ‘do you have problems with waking up still feeling tired?’, which was used to measure self-reported exhaustion. If the participant answered yes to the question, they were considered as having exhaustion. (3) Weakness: Grip strength in the non-dominant hand was measured with a dynamometer (Jamar TM Hidraulic Hand Dynamometer, Preston, Jackson, Missouri, EEUU) adjusted for body mass index (BMI) according to the lowest quintile. The cut-off points established were, for men: BMI ≤ 24 and grip strength < 18.5 kg; 24  <  BMI ≤ 28 and grip strength < 20 kg; BMI > 28 and grip strength < 22 kg; and for women: BMI ≤ 29 and grip strength < 11 kg; BMI > 29 and grip strength < 12 kg. (4) Slowness: Calculated after walking 3 m, adjusted for sex and height, according to Fried. The cut-off points for 3 m were established as, in men: height ≤ 173 cm and time ≥ 4.59 s (equivalent to 0.65 m/s); height > 1.73 cm and time ≥ 3.93 s (equivalent to 0.76 m/s); and for women: height ≤ 1.59 cm and time ≥ 4.59 s (0.65 m/s); and height > 1.59 cm and time ≥ 3.93 s (0.76 m/s). (5) Low physical activity: Calculated using a Physical Activity Questionnaire [20], which is used to record daily physical activity (walking, cycling, light and heavy household chores, and gardening) and physical exercise. If the individual scored 0 on any of the frailty components, they were considered as non-frail, if they scored between 1 and 2, they were considered as pre-frail, and if they scored 3 or more, they were considered frail [19].

### 2.4. Statistical Analysis

Data was analyzed using the Statistical Package for Social Sciences (SPSS) version 27.0. Continuous variables that are normally distributed were presented in terms of mean ± SD, while continuous variables that are not normally distributed were presented in terms of median interquartile range. Kolmogorov–Smirnov was used to test the normality of continuous variables. Categorical variables were presented in terms of frequency and percentage. One-way ANOVA was used to compare means between non-frail, pre-frail, and frail groups. The chi-square test was used to compare proportions between groups. Binary logistic regression with the backward condition was used to determine the association between frailty and associated factors (age, biological sex, chronic conditions, obesity, lifestyle factors, socio-economic factors, and lipid profile). A backward stepwise procedure was used to eliminate variables that were not statistically significant. This method fits the full model with all independent variables, then assesses the statistical significance of each predictor using the Wald test (*p*-values). Next, it removes the least significant predictor, with a *p* value over 0.05, and refits the model without the removed variable. This process is repeated until all remaining variables are statistically significant. Frailty was the dependent variable, while sociodemographic profile and biochemical profiles (lipids, glucose) were the independent variables. A *p*-value of less than 0.05 was considered statistically significant for all inferential statistics.

## 3. Results

### 3.1. Characteristics of Participants

Table 1 presents general characteristics of participants. The present study comprised 546 participants, of which 48.4% were males and 51.6% were females, with a mean age of 66.78 ± 5.72. Participants who were married accounted for 56.6% and 53.3% had primary school as their highest level of education. The frequency of chronic illnesses was as follows: Hypertension (41.3%), diabetes (13.6%), and HIV (10.7%). Approximately 32.5% of the participants were obese. The prevalence of individuals living with frailty was 26.4%.

### 3.2. Comparison of Means and Proportions of Sociodemographic and Biochemical Profiles Across the Frailty Groups (Non-Frail, Pre-Frail, and Frail)

Individuals living with frailty were significantly older than both pre-frail and non-frail (*p* < 0.001). Pre-frailty was more prevalent among women (59.1%), whereas frailty was more prevalent among men (63.9%), with a significant difference noted (*p* < 0.001). Current smokers had a higher proportion of frailty compared to both pre-frail and non-frail (*p* < 0.023). There was no significant difference between the three groups in terms of marital status (*p* = 0.146). The proportion of frailty reduced as the level of education increased. There was a significant difference in BMI between the non-frail and frail groups, with a *p*-value of <0.001. There was a significant difference in waist circumference between the frail and the non-frail group, with *p*-values of <0.001. There was a significant difference in subcutaneous fat in the non-frail and frail groups and between the frail and pre-frail groups (*p* < 0.049). Significantly more people who were obese fell into the pre-frail category, with the *p*-value of <0.004. There was a significant difference in LDL/HDL-c and TC/HDL-c lipid ratios between the frail and non-frail groups, with *p*-values of 0.043 and 0.040, respectively. There was no significant difference in systolic and diastolic blood pressure across the three groups, with respective *p*-values of 0.878 and 0.270. There was no significant difference in lipid profiles and glucose across the three groups (Table 2).

### 3.3. Factors Associated with Frailty

**Figure 1:**
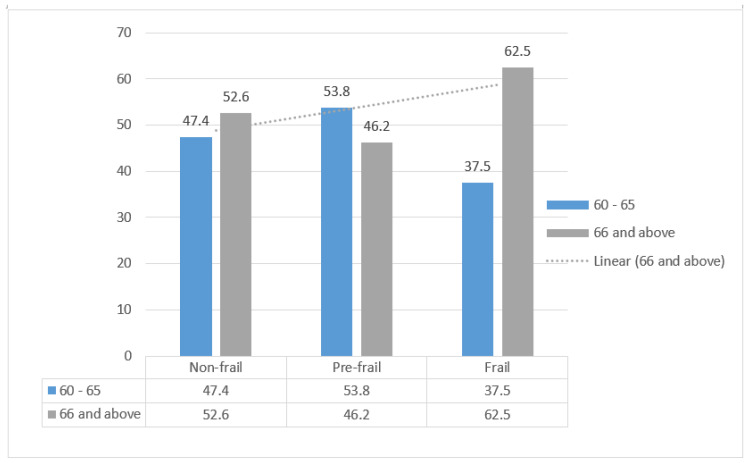
Differences in the proportion of frailty by age category.

Frail participants were significantly older. As the age of the participants increases, so does frailty, see Figure 1 below.

On the unadjusted model, participants aged 66 years and above were more likely to be frail compared to those aged 60–65 years (OR = 1.61; 95% CI: 1.00–2.60; *p* = 0.049). Central obesity was associated with significantly lower odds of frailty (OR = 0.48; 95% CI: 0.26–0.86; *p* = 0.040), while high total cholesterol was linked to increased frailty risk (AOR = 2.25; 95% CI: 1.04–4.88; *p* = 0.040). The present study found no significant association between biological sex, marital status, smoking, alcohol status, current smoking, hypertension, diabetes, and obesity with frailty. In the fully adjusted model, participants aged 66 years and above had significantly higher odds of being frail compared to those aged 60–65 years (AOR = 1.75; 95% CI: 1.4–2.70; *p* = 0.011). Central obesity was significantly associated with lower odds of frailty (AOR = 0.37; 95% CI: 0.24–0.57; *p* < 0.001), suggesting a potentially protective role. Additionally, high total cholesterol levels were positively associated with frailty (AOR = 2.05; 95% CI: 1.03–4.01; *p* = 0.041). In contrast, variables such as biological sex (female: OR = 0.71; 95% CI: 0.38–1.33; *p* = 0.282), marital status (e.g., married: OR = 1.53; 95% CI: 0.79–2.78; *p* = 0.206), education level, current alcohol consumption (OR = 1.03; 95% CI: 0.58–1.82; *p* = 0.917), smoking status (OR = 1.05; 95% CI: 0.55–2.01; *p* = 0.884), hypertension (OR = 0.89; 95% CI: 0.57–1.40; *p* = 0.609), diabetes (OR = 1.09; 95% CI: 0.53–2.24; *p* = 0.809), HIV status (OR = 0.93; 95% CI: 0.45–1.94; *p* = 0.848), and other lipid markers including high LDL-c (OR = 0.41; 95% CI: 0.18–0.91; *p* = 0.029), low HDL-c, and elevated lipid ratios (TC/HDL-c, TG/HDL-c, LDL/HDL-c) were not significantly associated with frailty in the adjusted model (refer to Table 3).

## 4. Discussion

The present study aimed to evaluate the prevalence of frailty and associated factors in older participants residing in the DIMAMO Surveillance area, Limpopo Province, South Africa. The proportion of individuals living with frailty was 24.6%, which is within the range of frailty in South Africa, reported to range between 4 and 59.1%, depending on the population sampled and method used [21,22]. A study by Biritwum et al. [8] reported a frailty prevalence of 30.8% in Ghana, which is higher than the prevalence observed in the current study but falls within the range reported for frailty in South Africa.

Individuals living with frailty were significantly older than both the pre-frail and non-frail. In logistic regression, with a backward condition, an increase with age was associated with frailty. In agreement with the findings of the present study, previous studies reported that an increase in age is associated with frailty. This may be because older people have an increased vulnerability to age-related diseases such as diabetes mellitus and hypertension, which may affect their overall health and ultimately make older individuals more susceptible to frailty [23,24,25]. In addition, the high likelihood of frailty in this age group could be due to the existence of physiological deterioration, which is prevalent as they age [26,27,28,29,30].

The proportion of frailty was higher in males than females; however, the present study found no significant association between biological sex and frailty. There have been contradicting reports relating to the association of frailty with biological sex. For instance, some studies reported a higher prevalence of frailty in males than in females, caused by higher engagement in high-risk behaviors and characterized by hand-grip strength loss, exhaustion, and low physical activity [31,32]. Older males are reported to have high levels of physiological dysregulation and lower body mass coupled with skeletal muscle loss, which contributes to frailty [33,34]. On the other hand, several studies reported the prevalence of frailty to be more prevalent in women than men, despite women having longer life expectancy and lower specific mortality rates per age than men [21,35]. In addition, Palomo et al. [31], reported that the prevalence of individual frailty components such as weakness, weight loss, slowness, exhaustion, depressive symptoms, and low physical activity was more prevalent in women than men. The high prevalence of frailty and its components in women was attributed to frailty risk factors such as abdominal obesity, which was exacerbated in the group of older women [36]. Physical frailty has also been associated with late-life depression (more prevalent in older women), which further explains the reported high prevalence of frailty in women [37]. The above literature indicates that the mechanism for frailty is different between males and females, thus suggesting gender specific interventions for frailty in older individuals.

The findings of the present study noted that the proportion of frailty to reduce as the level of education increased. In alignment with the findings of the present study, previous studies have indicated that lower educational attainment is linked with a higher risk of frailty, whereas higher education appears to serve as a protective factor [38,39]. This could be that individuals with higher educational levels are more informed about healthy lifestyle choices, maintain a well-balanced diet, and are more likely to engage in regular physical activity [40,41].

The present study found no association between smoking and alcohol consumption with frailty. In contrast with the findings of the present study, DeClercq et al. [42], reported a positive association between alcohol consumption and smoking with frailty. The difference between the findings of the present study and a study by DeClercq et al. [42] may be due to that the present study used self-reported data on both alcohol and smoking, which may have some limitations or potential bias that can lead to misclassification and weaken the observed associations.

Previous studies have reported both positive and negative associations between marital status and frailty, whereby married participants were reported to be at less risk of being frail as compared to those who never married, divorced, and widowed, due to loneliness and lack of emotional support [43,44]. Conversely, a study by Boucham et al. [45] have noted no significant association between marital status and frailty, even after adjusting for other factors such as income, education, and lifestyle factors. In agreement with Boucham et al. [45] the present study found that there was no significant association between frailty and marital status. This suggests that although marital status may have an effect on frailty, it may not be the sole determinant of frailty.

Previous studies have reported central obesity to be associated with frailty [46,47], thus demonstrating central obesity as a better biomarker in assessing frailty, especially in older people. In contrast, in the present study, central obesity was reported no be a protective barrier against frailty. The inconsistencies between the present study and previous studies were the difference in age groups; previous studies enrolled participants aged 60 and above, while the present study enrolled participants aged 40 and above. Moreover, there was no difference in geographical locations.

In the present study, dyslipidemia was positively associated with frailty. Binary logistic regression reported elevated TC/HDL-c to be positively associated with frailty. In agreement with the present study, Shakya et al. [48], and Ma et al. [49], reported dyslipidemia to be associated with the development of frailty. An elevated TC/HDL-c ratio is often associated with insulin resistance and metabolic dysfunction [50,51]. These conditions can impair glucose uptake by cells and disrupt energy metabolism, potentially contributing to muscle wasting, weakness, and frailty [52,53]. In addition, dyslipidemia, particularly high triglyceride levels, can lead to inflammation and oxidative stress in the body [54]. Chronic inflammation and oxidative stress are known to contribute to the development of frailty by promoting tissue damage and impairing physiological functions [54].

### Limitations of the Study

The findings of the present study should be interpreted with the following limitations. Firstly, the lack of representativeness: the sampling method used in this study may limit its generalizability due to the potential for selection bias, as certain demographic groups may be overrepresented or underrepresented in the sample. Secondly, due to the sampling method utilized, it was not feasible to ascertain the true prevalence of the phenomenon under investigation. Consequently, proportions are reported instead, which may not accurately reflect the prevalence of the frailty within the target population. Lastly, the cross-sectional design of the study poses limitations in establishing causal relationships between frailty and biomedical factors. As data is collected at a single point in time, temporal sequencing and causality cannot be inferred. This hinders the ability to determine whether frailty influences biomedical factors, vice versa, or if there are other underlying factors contributing to observed associations.

## 5. Conclusions

The proportion of frailty in the present study was 26.4%. Frailty was determined by age, educational status, and dyslipidemia. Based on the findings of the present study, the authors recommend implementation of screening programs for frailty in healthcare settings, especially targeting older adults and individuals with comorbidities such as obesity, and dyslipidemia. Regular screening can help in early identification and intervention. Moreover, we encourage additional research aimed at enhancing our comprehension of the fundamental mechanisms behind frailty and investigating novel approaches to prevent and manage it. By promoting collaboration among researchers, clinicians, and policymakers, we can improve the progress in frailty care, leading to better outcomes for those suffering from it.

## Figures and Tables

**Table 1:** General characteristics of participants.

Variables		*N* (%)/Mean ± SD
**Age**		66.78 ± 5.72
**Biological sex**	Males	264 (48.40)
	Females	282 (51.6)
**Marital status**	Single	92 (16.9)
	Married	310 (56.9)
	Divorced	52 (9.50)
	Widowed	91 (16.7)
**Educational status**	No formal education	57 (10.4)
	Primary	291 (53.3)
	Secondary	184 (33.7)
	Tertiary	14 (2.60)
**Current smoker**		125 (22.9)
**Current alcohol consumption**		168 (30.8)
**Hypertension**		225 (41.3)
**Diabetes**		72 (13.6)
**Obesity**		176 (32.5)
**Central obesity**		336 (62.1)
**HIV**		55 (10.7)
**Frailty**		144 (26.4)

**Table 2:** Comparison of sociodemographic and biochemical profiles between non-frail, pre-frail, and frail.

		Non-Frail	Pre-Frail	Frail	*p*-Value
**Age (mean ± SD)**		65.6 ± 3.76	66.02 ± 5.09	68.94 ± 6.88	<0.001 ^bc^
**Biological sex**	Male *N* (%)	4 (21.1)	168 (43.9)	92 (63.9)	<0.001
	Female *N* (%)	15 (78.9)	215 (56.1)	52 (36.1)	
**Marital status**	Single *N* (%)	7 (36.8)	62 (16.2)	23 (16.1)	0.146
	Married *N* (%)	9 (47.4)	213 (55.6)	88 (61.5)	
	Divorced *N* (%)	1 (5.30)	36 (9.40)	13 (10.5)	
	Widowed *N* (%)	2 (10.5)	72(18.8)	17 (11.9)	
**Highest level of education**	No-formal education *N* (%)	0 (0.0)	36 (9.40)	21 (14.6)	0.011
	Primary *N* (%)	14 (73.7)	36 (9.40)	81 (56.3)	
	Secondary *N* (%)	3 (15.8)	141 (36.8)	40 (27.8)	
	Tertiary *N* (%)	2 (10.5)	10 (2.60)	2 (1.40)	
**Current smoker *N* (%)**		2 (10.5)	79 (20.6)	44 (30.6)	0.023
**Current alcohol consumption *N* (%)**		5 (26.3)	108 (28.2)	55 (38.2)	0.078
**BMI (mean ± SD)**		28.85 ± 5.86	28.40 ± 6.72	25.14 ± 9.00	<0.001 ^c^
**WC (mean ± SD)**		91.07 ± 9.39	93.91 ± 12.48	88.35 ± 15.61	<0.001 ^c^
**SBP (mean ± SD)**		138.79 ± 16.41	135.98 ± 22.51	136.22 ± 26.69	0.878
**DBP (mean ± SD)**		82.89 ± 7.81	79.01 ± 11.89	78.22 ± 12.44	0.270
**VAT (mean ± SD)**		5.36 ± 1.55	5.76 ± 2.36	5.31 ± 2.51	0.156
**SAT (mean ± SD)**		2.07 ± 0.82	1.84 ± 1.07	1.60 ± 1.05	0.049
**Glucose (mean ± SD)**		5.52 ± 0.75	5.99 ± 2.21	5.89 ± 2.29	0.622
**HDL-c (mean ± SD)**		1.29 ± 0.24	1.26 ± 0.35	1.34 ± 0.44	0.067
**LDL-c (mean ± SD)**		3.01 ± 0.83	2.84 ± 0.93	2.67 ± 0.82	0.109
**TC (mean ± SD)**		4.93 ± 0.96	4.72 ± 1.10	4.60 ± 1.01	0.358
**TG (mean ± SD)**		1.38 ± 0.58	1.34 ± 0.58	1.29 ± 0.68	0.721
**LDL/HDL-c (mean ± SD)**		2.39 ± 0.74	2.36 ± 0.83	2.16 ± 0.84	0.043 ^c^
**TC/HDL-c (mean ± SD)**		3.89 ± 0.86	3.91 ± 0.98	3.65 ± 1.04	0.040 ^c^
**TG/HDL-c (mean ± SD)**		1.11 ± 0.54	1.16 ± 0.65	1.10 ± 0.75	0.593
**Obesity *N* (%)**		8 (42.1)	137 (36.2)	31 (21.5)	0.004
**Diabetes *N* (%)**		1 (5.3)	54 (14.5)	17(12.6)	0.451
**Hypertension *N* (%)**		9 (47.4)	156 (40.8)	60 (41.7)	0.848
**HIV *N* (%)**		0 (0.0)	43 (11.8)	12 (9.0)	0.223
**Central obesity *N* (%)**		14 (73.3)	256 (67.7)	66 (45.8)	<0.001

Data were presented in terms of mean ± standard deviation or number (%). *p*-value: significance of the results (*p* < 0.05). BMI: body mass index, SBP: systolic blood pressure, DBP: diastolic blood pressure, HDL-C: high-density lipoproteins cholesterol. LDL-C: low-density lipoprotein cholesterol. TC: total cholesterol. TG: triglycerides: non-frail vs. frail = ^b^, frail vs. pre-frail = ^c^.

**Table 3:** Multivariate logistic regression with a backward condition of frailty associated with sociodemographic profiles and biochemical profiles.

Variables	Unadjusted Model	Fully Adjusted Model			
		Exp B (95% CI)	*p* Value	Exp B (95% CI)	*p* Value
**Biological sex**	Male	ref			
	Female	0.71 (0.38–1.33)	0.282		
**Age groups**	60 to 65	ref			
	66 and above	1.61 (1.00–2.60)	0.049	1.75 (1.4–2.70)	0.011
**Marital status**	Married	1.53 (0.79–2.78)	0.206		
	Single	1.68 (0.75–3.75)	0.205		
	Divorced	1.60 (0.66–3.86)	0.292		
	Widowed	ref			
**Highest level of education**	No formal education	ref			
	Primary	0.79 (0.39–1.60)	0.509		
	Secondary	0.61(0.28–1.30)	0.198		
	Tertiary	0.31(0.03–2.94)	0.309		
**Current alcohol consumption**	No	ref			
	Yes	1.03(0.58–1.82)	0.917		
**Current smoker**	No	ref			
	Yes	1.05 (0.55–2.01)	0.884		
**Hypertension**	No	ref			
	Yes	0.89 (0.57–1.40)	0.609		
**Diabetes**	No	ref			
	Yes	1.09 (0.53–2.24)	0.809		
**Obesity**	No	ref			
	Yes	1.02 (0.55–1.87)	0.959		
**Central obesity**	No	ref			
	Yes	0.48 (0.26–0.86)	0.040	0.37 (0.24–0.57)	<0.001
**HIV**	Negative	ref			
	Positive	0.93 (0.45–1.94)	0.848		
**High Trig**	No	ref			
	Yes	0.69 (0.36–1.33)	0.268		
**High TC**	No	Ref			
	Yes	2.25 (1.04–4.88)	0.040	2.05 (1.03–4.01)	0.041
**High LDL-c**	No	ref			
	Yes	0.41 (0.18–0.91)	0.029		
**Low HDL-c**	No	ref			
	Yes	0.83 (0.47–1.48)	0.526		
**High TC/HDL-c**	No	ref			
	Yes	1.85 (0.76–4.52)	0.179		
**High TG/HDL-c**	No	ref			
	Yes	4.55 (0.27–76.3)	0.292		
**High LDL/HDL-c**	No	ref			
	Yes	1.35 (0.77–2.35)	0.296		

Adjusted for age, biological sex, chronic conditions, obesity, lifestyle factors, socio-economic factors, and lipid profile.

## Data Availability

The data presented in this study is available on request from the corresponding author. The data is not publicly available due to privacy or ethical restrictions.

## Abbreviations

DIMAMO	Dikgale Mamabolo Mothiba

## References

[1] Proietti M., Cesari M., Veronese N. (2020). Frailty: What Is It?. Frailty and Cardiovascular Diseases: Research into an Elderly Population.

[2] British Geriatrics Society Introduction to Frailty. https://www.bgs.org.uk/resources/introduction-to-frailty.

[3] Database.earth Life Expectancy of South Africa 1950–2024 & Future Projections. https://database.earth/population/South-Africagrowth-rate.

[4] De Wet-Billings N. (2021). Preventable deaths among youth in South Africa: Measuring life expectancy in the absence of non-communicable diseases and its implications for the healthcare system. S. Afr. Med. J..

[5] Maredza M., Bertram M.Y., Gómez-Olivé X.F., Tollman S.M. (2016). Burden of stroke attributable to selected lifestyle risk factors in rural South Africa. BMC Public Health.

[6] Lai H.Y., Huang S.T., Anker S.D., von Haehling S., Akishita M., Arai H., Chen L., Hsiao F. (2024). The burden of frailty in heart failure: Prevalence, impacts on clinical outcomes and the role of heart failure medications. J. Cachexia Sarcopenia Muscle.

[7] George N.L., Nethathe G.D. (2019). Frailty: What the South African surgeon needs to know. S. Afr. J. Surg..

[8] Biritwum R.B., Minicuci N., Yawson A.E., Theou O., Mensah G.P., Naidoo N., Wu F., Guo Y., Zheng Y., Jiang Y. (2016). Prevalence of and factors associated with frailty and disability in older adults from China, Ghana, India, Mexico, Russia and South Africa. Maturitas.

[9] Weiss C.O. (2011). Frailty and chronic diseases in older adults. Clin. Geriatr. Med..

[10] Onder G., Vetrano D.L., Marengoni A., Bell J.S., Johnell K., Palmer K. (2018). Accounting for frailty when treating chronic diseases. Eur. J. Intern. Med..

[11] Ntimana C.B., Mashaba R.G., Seakamela K.P., Netshapapame T., Maimela E. (2023). Risky sexual behaviors and associated factors among adult patients on antiretroviral treatment at Mankweng Hospital in Limpopo Province, South Africa. Front. Public Health.

[12] Maimela E., Alberts M., Modjadji S.E., Choma S.S., Dikotope S.A., Ntuli T.S., Van Geertruyden J.-P., Oni T. (2016). The prevalence and determinants of chronic non-communicable disease risk factors amongst adults in the Dikgale health demographic and surveillance system (HDSS) site, Limpopo Province of South Africa. PLoS ONE.

[13] Ringane M.C., Choma S.S.R. (2021). The optimal WC cut-off points for the prediction of subclinical CVD as measured by carotid intima-media thickness among African adults: A cross-sectional study. BMC Cardiovasc. Disord..

[14] Moshidi M.L., Malema R.N., Muthelo L., Mothiba T.M. (2021). Provision of Care to the People with HIV: Voices of Professional Nurses in the Public Hospitals of Limpopo Province, South Africa. Int. J. Environ. Res. Public Health.

[15] Conan N., Simons E., Chihana M.L., Ohler L., FordKamara E., Mbatha M., Vancutsem G., Huerga H., Parekh B.S. (2022). Increase in HIV viral suppression in KwaZulu-Natal, South Africa: Community-based cross sectional surveys 2018 and 2013. What remains to be done?. PLoS ONE.

[16] Ntimana C.B., Choma S.S. (2023). Modifiable determinants of central obesity among the rural black population in the DIMAMO HDSS, Limpopo, South Africa. Front. Public Health.

[17] Ali S.A., Soo C., Agongo G., Alberts M., Amenga-Etego L., Boua R.P., Choudhury A., Crowther N.J., Depuur C., Gómez-Olivé F.X. (2018). Genomic and environmental risk factors for cardiometabolic diseases in Africa: Methods used for Phase 1 of the AWI-Gen population cross-sectional study. Glob. Health Action.

[18] Ntimana C.B., Mashaba R.G., Seakamela K.P., Maimela E., Masemola-Maphutha M.L., Choma S.S. (2024). Comorbidities of Obesity in a Rural African Population Residing in Limpopo Province, South Africa: A Comparison between General and Central Obesity. Obesities.

[19] Fried L.P., Ferrucci L., Darer J., Williamson J.D., Anderson G. (2004). Untangling the concepts of disability, frailty, and comorbidity: Implications for improved targeting and care. J. Gerontol. A Biol. Sci. Med. Sci..

[20] Herrmann S.D., Heumann K.J., Der Ananian C.A., Ainsworth B.E. (2013). Validity and Reliability of the Global Physical Activity Questionnaire (GPAQ). Meas. Phys. Educ. Exerc. Sci..

[21] Payne C.F., Wade A., Kabudula C.W., Davies J.I., Chang A.Y., Gomez-Olive F.X., Kahn K., Berkman L.F., Tollman S.M., Salomon J.A. (2017). Prevalence and correlates of frailty in an older rural African population: Findings from the HAALSI cohort study. BMC Geriatr..

[22] Kasa A.S., Lee S.C., Chang H.C.R. (2024). Frailty in older people living in Africa: A systematic review of prevalence and associated factors. Arch. Gerontol. Geriatr. Plus.

[23] Soto M.E., Pérez-Torres I., Rubio-Ruiz M.E., Cano-Martínez A., Manzano-Pech L., Guarner-Lans V. (2023). Frailty and the Interactions between Skeletal Muscle, Bone, and Adipose Tissue-Impact on Cardiovascular Disease and Possible Therapeutic Measures. Int. J. Mol. Sci..

[24] Gielen E., Dupont J., Dejaeger M., Laurent M.R. (2023). Sarcopenia, osteoporosis and frailty. Metabolism.

[25] Lee A., McArthur C., Ioannidis G., Duque G., Adachi J.D., Griffith L.E., Thabane L., Papaioannou A. (2024). Associations between Osteosarcopenia and Falls, Fractures, and Frailty in Older Adults: Results From the Canadian Longitudinal Study on Aging (CLSA). J. Am. Med. Dir. Assoc..

[26] Grainger S.A., Crawford J.D., Riches J.C., Kochan N.A., Chander R.J., Mather K.A., Sachdev P.S., Henry J.D., Krendl A. (2023). Aging Is Associated With Multidirectional Changes in Social Cognition: Findings From an Adult Life-Span Sample Ranging From 18 to 101 Years. J. Gerontol. Ser. B.

[27] Bauman A., Merom D., Bull F.C., Buchner D.M., Fiatarone Singh M.A. (2016). Updating the Evidence for Physical Activity: Summative Reviews of the Epidemiological Evidence, Prevalence, and Interventions to Promote “Active Aging”. Gerontologist.

[28] Rantakokko M., Mänty M., Rantanen T. (2013). Mobility decline in old age. Exerc. Sport Sci. Rev..

[29] Piche E., Chorin F., Gerus P., Jaafar A., Guerin O., Zory R. (2023). Effects of age, sex, frailty and falls on cognitive and motor performance during dual-task walking in older adults. Exp. Gerontol..

[30] Mitnitski A., Collerton J., Martin-Ruiz C., Jagger C., von Zglinicki T., Rockwood K., Kirkwood T.B.L. (2015). Age-related frailty and its association with biological markers of ageing. BMC Med..

[31] Isernia S., Cazzoli M., Baglio G., Cabinio M., Rossetto F., Giunco F., Baglio F., Blasi V. (2023). Differential roles of neural integrity, physical activity and depression in frailty: Sex-related differences. Brain Sci..

[32] Gordon E.H., Hubbard R.E. (2020). Differences in frailty in older men and women. Med. J. Aust..

[33] Cohen A.A., Legault V., Li Q., Fried L.P., Ferrucci L. (2018). Men sustain higher dysregulation levels than women without becoming frail. J. Gerontol. Ser. A.

[34] Lu Y.W., Chang C.C., Chou R.H., Lee W.J., Chen L.K., Huang P.H., Lin S.-J. (2024). Sex differences in the frailty phenotype and mortality in the I-Lan longitudinal aging study cohort. BMC Geriatr..

[35] O’Caoimh R., Sezgin D., O’Donovan M.R., Molloy D.W., Clegg A., Rockwood K., Liew A. (2021). Prevalence of frailty in 62 countries across the world: A systematic review and meta-analysis of population-level studies. Age Ageing.

[36] Palomo I., García F., Albala C., Wehinger S., Fuentes M., Alarcón M., Arauna D., Montecino H., Mendez D., Sepúlveda M. (2022). Characterization by Gender of Frailty Syndrome in Elderly People according to Frail Trait Scale and Fried Frailty Phenotype. J. Pers. Med..

[37] Brown P.J., Roose S.P., O’Boyle K.R., Ciarleglio A., Maas B., Igwe K.C., Chung S., Gomez S., Naqvi M., Brickman A.M. (2020). Frailty and Its Correlates in Adults With Late Life Depression. Am. J. Geriatr. Psychiatr..

[38] Santamaría-Ulloa C., Lehning A.J., Cortés-Ortiz M.V., Méndez-Chacón E. (2023). Frailty as a predictor of mortality: A comparative cohort study of older adults in Costa Rica and the United States. BMC Public Health.

[39] Feng Z., Lugtenberg M., Franse C., Fang X., Hu S., Jin C., Raat H., Ginsberg S.D. (2017). Risk factors and protective factors associated with incident or increase of frailty among community-dwelling older adults: A systematic review of longitudinal studies. PLoS ONE.

[40] Ntimana C.B., Seakamela K.P., Mashaba R.G., Maimela E. (2024). Determinants of central obesity in children and adolescents and associated complications in South Africa: A systematic review. Front. Public Health.

[41] Mashala D.G., Ntimana C.B., Seakamela K.P., Mashaba R.G., Maimela E. (2024). Sociodemographic Disparities in the Prevalence of Metabolic Syndrome in Rural South Africa: An Analysis of Gender, Age, and Marital, Employment, and Educational Status. Obesities.

[42] DeClercq V., Duhamel T.A., Theou O., Kehler S. (2020). Association between lifestyle behaviors and frailty in Atlantic Canadian males and females. Arch. Gerontol. Geriatr..

[43] Trevisan C., Grande G., Vetrano D.L., Maggi S., Sergi G., Welmer A.K., Rizzuto D. (2020). Gender Differences in the Relationship Between Marital Status and the Development of Frailty: A Swedish Longitudinal Population-Based Study. J. Womens Health.

[44] Kojima G., Walters K., Iliffe S., Taniguchi Y., Tamiya N. (2020). Marital Status and Risk of Physical Frailty: A Systematic Review and Meta-analysis. J. Am. Med. Dir. Assoc..

[45] Boucham M., Salhi A., El Hajji N., Gbenonsi G.Y., Belyamani L., Khalis M. (2024). Factors associated with frailty in older people: An umbrella review. BMC Geriatr..

[46] Crow R.S., Lohman M.C., Titus A.J., Cook S.B., Bruce M.L., Mackenzie T.A., Bartels S.J., Batsis J.A. (2019). Association of Obesity and Frailty in Older Adults: NHANES 1999–2004. J. Nutr. Health Aging.

[47] Newman A.B., Enright P.L., Manolio T.A., Haponik E.F., Wahl P.W., On behalf of the Cardiovascular Health Study Research Group (1997). Sleep Disturbance, Psychosocial Correlates, and Cardiovascular Disease in 5201 Older Adults: The Cardiovascular Health Study. J. Am. Geriatr. Soc..

[48] Shakya S., Bajracharya R., Ledbetter L., Cary M.P. (2022). The association between cardiometabolic risk factors and frailty in older adults: A systematic review. Innov. Aging.

[49] Ma L.L., Chen N., Zhang Y., Feng X.M., Gong M., Yan Y.X. (2024). Association of phenotypic frailty and frailty index with type 2 diabetes and dyslipidemia in middle-aged and elderly Chinese: A longitudinal cohort study. Arch. Gerontol. Geriatr..

[50] Xu L., Zhang J., Shen S., Hong X., Zeng X., Yang Y., Liu Z., Chen L., Chen X. (2020). Association Between Body Composition and Frailty in Elder Inpatients. Clin. Interv. Aging.

[51] Chandrasekaran P., Weiskirchen R. (2024). The Role of Obesity in Type 2 Diabetes Mellitus—An Overview. Int. J. Mol. Sci..

[52] Jung U.J., Choi M.S. (2014). Obesity and its metabolic complications: The role of adipokines and the relationship between obesity, inflammation, insulin resistance, dyslipidemia and nonalcoholic fatty liver disease. Int. J. Mol. Sci..

[53] Wu H., Ballantyne C.M. (2020). Metabolic Inflammation and Insulin Resistance in Obesity. Circ. Res..

[54] Inglés M., Gambini J., Carnicero J.A., García-García F.J., Rodríguez-Mañas L., Olaso-González G., Dromant M., Borrás C., Viña J. (2014). Oxidative Stress Is Related to Frailty, Not to Age or Sex, in a Geriatric Population: Lipid and Protein Oxidation as Biomarkers of Frailty. J. Am. Geriatr. Soc..

